# Evaluation and quantification of pulmonary hyperinflation in three gravitational zones of domestic felines by computed tomography

**DOI:** 10.1186/cc13477

**Published:** 2014-03-17

**Authors:** A Rodrigues de Carvalho Martins, D Tabacchi Fantoni, D Aya Otsuki, A Magalhäes Ambrosio, A Brandäo de Campos Fonseca Pinto, J França dos Santos, C Rodrigues de Carvalho Martins, L Sá Malbouisson

**Affiliations:** 1 Faculdade de Medicina da Universidade de Sao Paulo, Brazil; 2 Faculdade de Medicina Veterinaria e Zootecnia da Universidade de Sao Paulo, Brazil; 3 UFAPE, Sao Paulo, Brazil

## Introduction

Mechanical ventilation (MV) aims to enhance blood oxygenation and to remove carbon dioxide. However, excessive hyperinflation by MV may cause lung injury.

## Methods

Eighteen cats (4 ± 1 kg) were anesthetized with propofol (loading dose of 6 mg/kg and constant rate infusion of 0.5 mg/kg/ minute) and neuromuscular blockade was achieved with rocuronium at 1 mg/kg/minute. Their lungs were initially mechanically ventilated in FiO_2 _of 40%, with peak inspiratory pressure (Ppeak) of 5 cmH_2_O for 20 minutes, and then the Ppeak was increased by 5 cmH_2_O increments until 15 cmH_2_O every 5 minutes. Following that, Ppeak was decreased by 2 cmH_2_O every 5 minutes until reaching Ppeak of 5 cmH_2_O. The ventilator maintained the respiratory rate and inspiratory time at 15 breaths/minute and 1 second, respectively. Between the Ppeak increments, we applied a 4-second pause for a 5-mm computed tomography (CT) scan of the thorax area. The radiographic attenuation (in Hounsfield units, HU) was classified as over-insufflation (1,000 to 900 HU), normal insufflation (900 to 500 HU) and atelectasic (500 to 100 HU). We split the lungs into three proportional gravitational zones (I, II and Ill) from apex to base.

## Results

The three zones presented increased over-insufflated areas and decreased areas with normal insufflation with increasing Ppeak from 5 to 15 cmH_2_O. At 5 cmH_2_O, the areas of over-insufflation and normal insufflation in zones I, II and Ill were 13% and 36%; 4% and 22%; and 0.7% and 15%, respectively. At 15 cmH_2_O, the areas of over-insufflation and normal insufflation in zones I, II and Ill were 74% and 55%; 81% and 57%; and 82% and 71%, respectively.

## Conclusion

The higher proportion of overly distended pulmonary areas in high Ppeak may increase the risk of lung injury. The lowest Ppeak (5 cmH_2_O) showed less potential to lung injury as it has higher areas of normal insufflation and less areas of over-insufflation in all gravitational zones.

